# Achieving Autism Accreditation in Cat A Prison

**DOI:** 10.1192/bjo.2022.118

**Published:** 2022-06-20

**Authors:** Rachel Daly, Kimbia Motherskill

**Affiliations:** 1 Oxleas NHS Foundation Trust, London, United Kingdom; 2 Oxleas NHS foundation trust, London, United States Minor Outlying Islands

## Abstract

**Aims:**

To ensure autististic prisoners are understood and receive necessary support in custodial environment.

**Methods:**

1. Prison healthcare staff and discipline staff jointly trained about autism and how it is best managed in prison setting.
2. Promotion re-education aids for prisons visually and verbally.
3. Prison staff as autistic champions.
4. Accessible autistic spectrum lead in healthcare team to coordinate need.

**Results:**

priority that prison becomes autism accredited by national autistic society in progress.

**Conclusion:**

There is increase of prisoners with neurodevelopmental disorders and ensuring their needs met in prison and this is CAT A challenging prison environment.

